# Intensive care unit admissions with and without COVID-19 in Finland from 2017 to 2021: a retrospective register-based study

**DOI:** 10.1186/s12871-023-02207-9

**Published:** 2023-07-24

**Authors:** Saara Jäntti, Ville Ponkilainen, Ilari Kuitunen, Mikko M. Uimonen, Tuomas Huttunen, Ville M. Mattila

**Affiliations:** 1 Faculty of Medicine and Health Technology, Tampere University, Kauppi Campus, Arvo Ylpön katu 34, Tampere, 33520 Finland; 2 Department of Surgery, Central Finland Hospital Nova, Hoitajantie 3, 40620 Jyväskylä, Finland; 3 Mikkeli Central Hospital, Porrassalmenkatu 35-37, Mikkeli, 50100 Finland; 4 School of Medicine, University of Eastern Finland, Yliopistonranta 1, Kuopio, 70211 Finland; 5 Department of Cardiothoracic Anesthesia, Tampere Heart Hospital, Teiskontie 35, Tampere, 33521 Finland; 6 Department of Orthopaedics and Traumatology, Tampere University Hospital, Teiskontie 35, Tampere, PL2000, 33521 Finland

**Keywords:** COVID-19, Intensive care unit, Critical care

## Abstract

**Background:**

After the COVID-19 pandemic started, critical care resources were expanded in Finland to manage a possible surge in patients requiring intensive care. The aim of this study was to evaluate the incidence of overall ICU admissions, patient diagnoses, characteristics, and length of stay during the pandemic.

**Methods:**

This retrospective hospital register-based study was conducted in two large and one mid-size Finnish public hospitals. The required data were collected from ICU patient information systems and all adult patients were included. Monthly and yearly incidences with 95% confidence intervals (CI) were counted per 100 000 persons-years by Poisson exact method and compared by incidence rate ratios (IRR).

**Results:**

A total of 4407 admissions to ICUs for any cause occurred during 2020. In 2021, this figure was 4931. During the reference years (2017–2019), the mean number of admissions to ICU was 4781. In 2020 and 2021, the proportion of patients requiring intensive care due to COVID-19 was only 3%. The incidence of all-cause ICU admissions decreased during the lockdown in 2020 when compared to the reference years. Before the start of the lockdown in February 2020, the IRR of all-cause ICU admissions was 1.02 (CI: 0.89 to 1.18). During the lockdown period, however, the IRR of all-cause ICU admissions decreased to 0.78 (CI: 0.67 to 0.90) in March. When the lockdown ended, the incidence rebounded to the same level as before the lockdown. However, in 2021, the incidence of ICU admissions remained at the same level when compared to the reference years. The most prominent changes occurred in the incidence of diseases of the nervous system, which includes epilepsy and seizures and transient cerebral ischemic attacks, in diseases of the respiratory system, and neoplasms.

**Conclusions:**

According to the findings of this study, the incidence of all-cause ICU admissions decreased after the lockdown was implemented in 2020. Furthermore, the percentage of patients requiring intensive care due to COVID-19 in Finland was only 3% in 2020 and 2021. These findings may serve to help in the planning and allocating of ICU resources during future pandemics.

## Background

During the COVID-19 pandemic, ICUs worldwide have faced numerous challenges. The increasing number of COVID-19 patients and a high number of patients requiring respiratory support, in addition to other patients requiring ICU treatment, have placed high demands on ICU capacity [1]. At the start of the pandemic in Finland, critical care resources were expanded to deal with a possible surge in COVID-19 patients requiring intensive care. The number of ICU admissions due to COVID-19 began to increase in March 2020. In Finland, the peak prevalence in ICU admissions in patients who tested positive for COVID-19 was 1.5 per 100 000 in April 2020. The largest number of patients needing ICU treatment was 83 patients on April 7, 2020. Thereafter, the prevalence remained below 0.90 per 100 000 (50 patients) during subsequent waves of the pandemic [2].

During the global outbreaks of SARS in 2002–2003 and a novel influenza H1N1 in 2009, a substantial increase in demand of ICU services was reported. According to previous research from New Zealand and Australia, during the H1N1 pandemic in winter 2009 the number of ICU admissions was 15 times the number of admissions due to viral pneumonitis in reference years. In 2002–2003, SARS pandemic created a strain on healthcare systems in Toronto. The supply of critical care staff was limited, and ICU beds were closed which caused limitation of beds for all critical ill patients [3, 4].

To our knowledge, the extent of the changes in overall ICU admissions and the length of ICU stay during the pandemic remains unclear. As the number of patients requiring intensive care due to COVID-19 has remained low during the pandemic in Finland, this is an important issue that needs to be addressed.

Previous literature has yielded conflicting results, as both increases and decreases in non-COVID-19 ICU admissions have been reported [5–7].

Therefore, the aim of this study was to evaluate the incidence of overall ICU admissions, patient demographics, and length of stay during the pandemic. Also, the number of confirmed COVID-19 patients in Finland requiring intensive care during the years 2020 and 2021 was assessed.

## Methods

This retrospective hospital register-based study was conducted in two large - Tampere University Hospital (tertiary level unit), and Central Finland Hospital (secondary level unit) and one mid-size - Mikkeli Central Hospital (secondary level) Finnish public hospitals. In total, these hospitals cover a catchment area of approximately 700 000 adult inhabitants (remained unchanged during the study years). During the study period, the mean age in Finnish population was 43 years. In the catchment area of Tampere University Hospital, the mean age of population was 43 years, in Mikkeli Central Hospital catchment area 49 years and in Central Finland Hospital catchment area 43 years. The distribution by gender was similar in all areas, 50% were men and 50% women [8]. The required data were collected from the ICU patient information systems of the three hospitals. All adult patients (aged 18 or older) who were admitted to the ICUs of the participating hospitals in 2020 and 2021 were included. As a reference, we used ICU admissions from 2017 to 2019. The classification of patients was done using International Classification of Diseases 10th Revision (ICD-10) diagnostic codes [9]. Data regarding ICU admission was collected using the first diagnosis of a patient and each treatment period was collected only once. The changes in the number of ICU admissions were collected and analyzed in categories based on the ICD-10 diagnostic codes (Table 1). The number of patients who were admitted to ICU and had tested positive for COVID-19 in the years 2020 and 2021 was collected using ICD-10 diagnostic codes starting with U07.1.

**Table 1:** The yearly number of ICU admissions and incidence in 2020 and 2021 compared to the mean yearly incidence of the reference years (2017–2019) by incidence rate ratios (IRR) with 95% confidence intervals

		2017–2019		2020				2021		
ICD code	Explanation	N	Inc	N	Inc	IRR (95% CI)		N	Inc	IRR (95% CI)
A. B	Infectious and parasitic diseases	273	37.8	266	36.6	0.97	(0.82–1.14)	250	34.2	0.90 (0.76–1.07)
C. D	Neoplasms. diseases of the blood	608	84.4	534	73.5	0.87	(0.78–0.98)	555	75.8	0.90 (0.80–1.01)
E	Endocrine. nutritional and metabolic diseases	647	89.7	591	81.3	0.91	(0.81–1.01)	602	82.3	0.92 (0.82–1.02)
F	Mental. Behavioral and Neurodevelopmental disorders	212	29.4	202	27.8	0.95	(0.78–1.15)	221	30.2	1.03 (0.85–1.24)
G	Diseases of the nervous system	219	30.4	194	26.7	0.88	(0.72–1.06)	352	48.1	1.58 (1.34–1.87)
I	Diseases of the circulatory system	1542	213.8	1429	196.7	0.92	(0.86–0.99)	1711	233.8	1.09 (1.02–1.17)
J	Diseases of the respiratory system	163	22.6	125	17.2	0.76	(0.60–0.96)	116	15.9	0.70 (0.55–0.89)
K	Diseases of the digestive system	81	11.2	114	15.7	1.40	(1.05–1.86)	74	10.1	0.90 (0.66–1.23)
M	Diseases of the musculoskeletal system and connective tissue	21	3.0	13	1.8	0.60	(0.30–1.21)	16	2.2	0.74 (0.39–1.41)
N	Diseases of the genitourinary system	17	2.4	20	2.8	1.17	(0.61–2.23)	22	3.0	1.28 (0.68–2.40)
O	Pregnancy. childbirth and the puerperium	19	2.6	18	2.5	0.94	(0.49–1.79)	18	2.5	0.93 (0.49–1.78)
S	Injury. poisoning and other external causes	221	30.7	238	32.8	1.07	(0.89–1.28)	196	26.8	0.87 (0.72–1.06)
T	Injury. poisoning and other external causes	174	24.2	168	23.1	0.96	(0.77–1.18)	220	30.1	1.24 (1.02–1.52)
U0*	Covid patients	0		110	15.1			141	19.3	
W	External causes of morbidity	437	60.6	372	51.2	0.84	(0.74–0.97)	390	53.3	0.88 (0.77–1.01)
R. H. L. Z	Other and unspecified	572	79.3	486	66.9	0.84	(0.75–0.95)	569	77.8	0.98 (0.87–1.10)

### Statistical analysis

Monthly and yearly incidences with 95% confidence intervals (CI) were counted per 100 000 person-years by Poisson exact method and compared by incidence rate ratios (IRR). Median and interquartile range (IQR) was calculated for ICU stay duration. The analyses and figures were performed using R version 3.6.2 (R Foundation for Statistical Computing, Vienna, Austria).

Ethics.

Due to the register-based study design, we did not obtain ethical committee evaluation. As the law on the secondary use of routinely collected health care data is rather strict, we did not combine the patient data from the participating hospitals. Instead, we analyzed the data from each hospital separately and then combined the data anonymously.

## Results

**Fig. 1:**
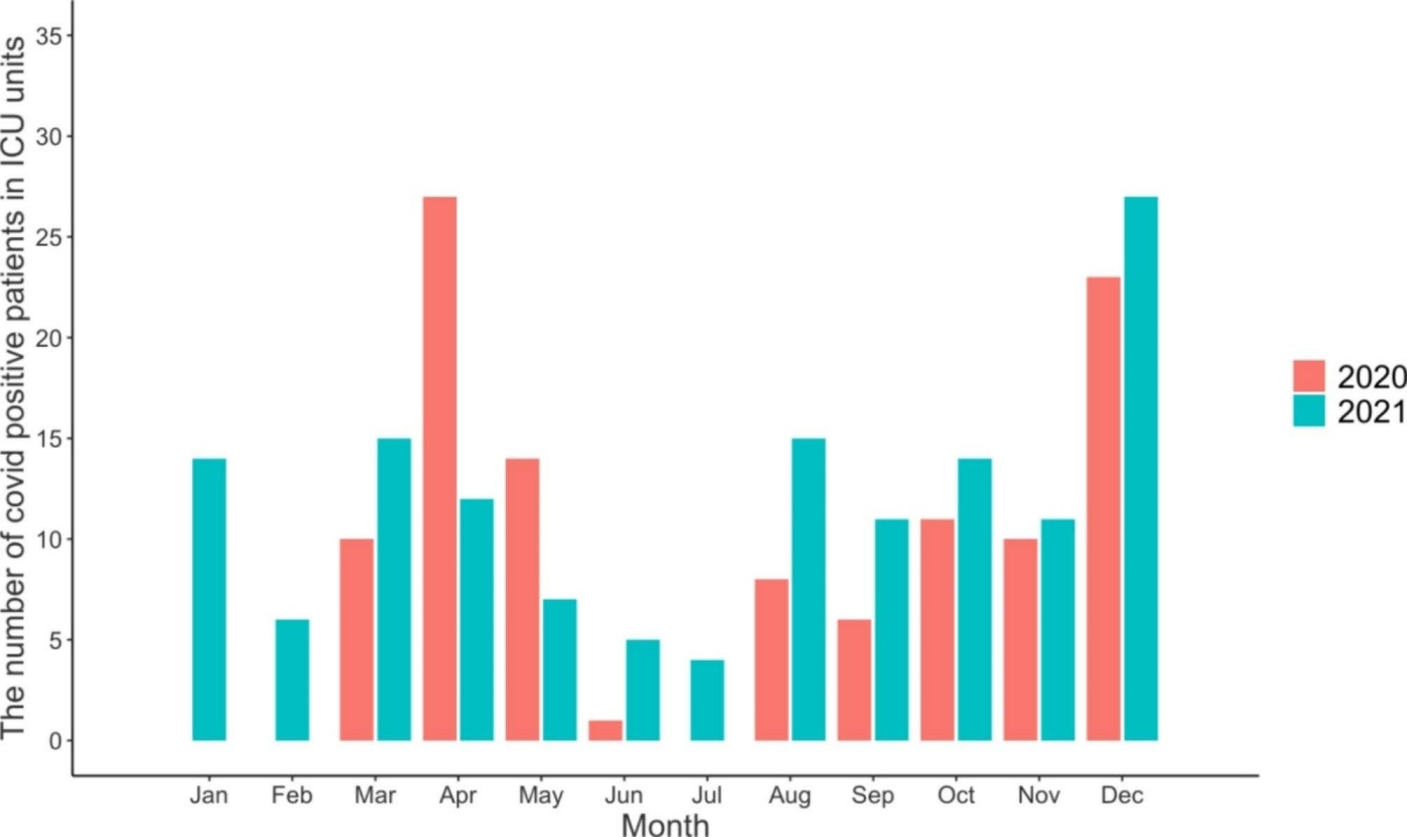
The number of COVID-positive patients in three ICU units during the COVID-19 pandemic

A total of 4407 admissions to ICU occurred in 2020 and 4931 in 2021. During the reference years (2017–2019), the mean number of admissions to ICU was 4781. A total of 4864 admissions occurred in 2017, 4856 in 2018, and 4624 in 2019. In addition, a total of 110 (2.5% of all ICU admissions) COVID-positive patients were admitted to ICU units in the participating hospitals in 2020. During the year 2021, the number of COVID patients admitted was 141 (2.9% of all ICU admissions). We found three distinctive peaks of COVID patients being admitted to ICU: 27 patients in April 2020, 23 patients in December 2020, and 27 patients in December 2021 (Fig. 1).

**Fig. 2:**
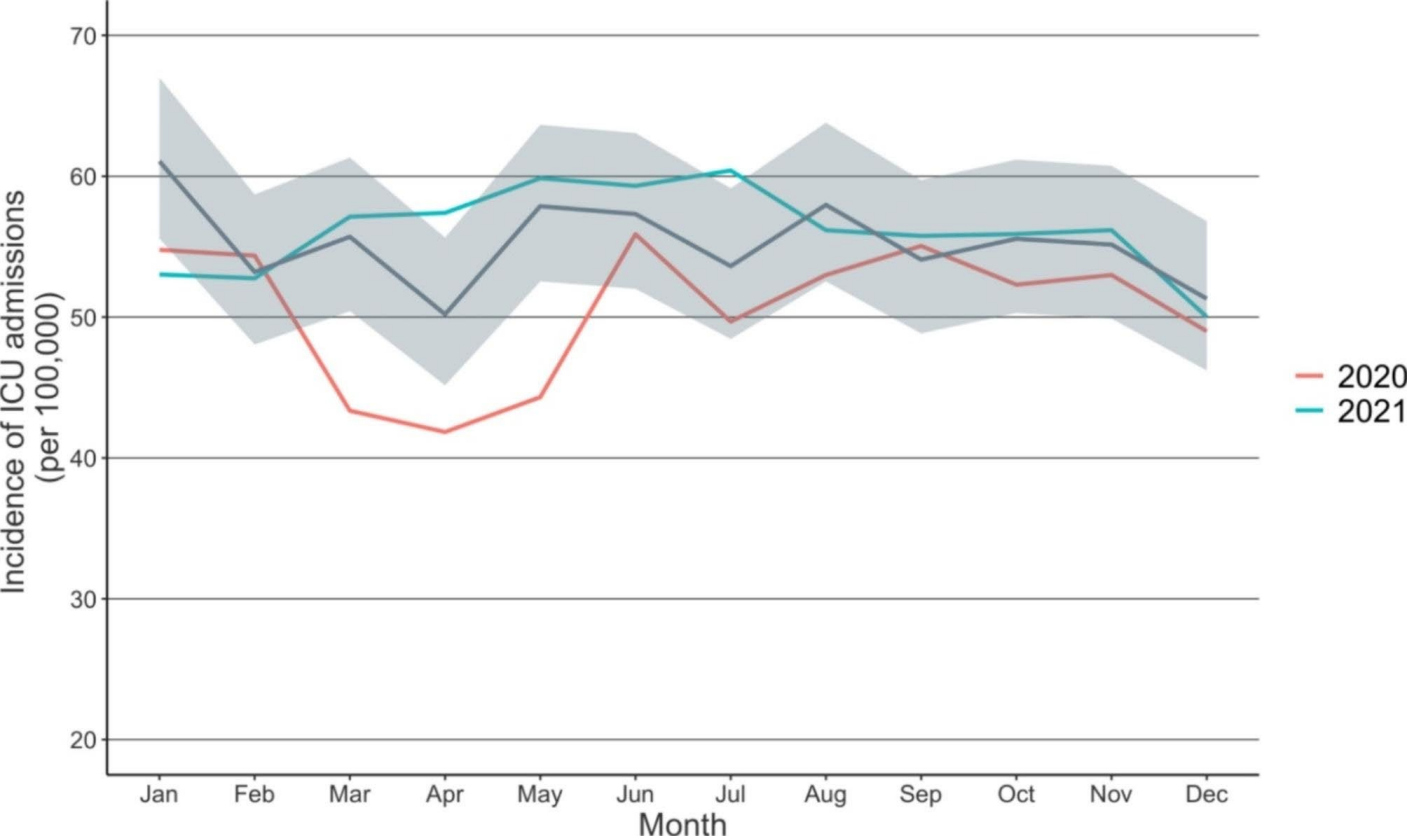
Incidence of ICU admissions for any cause during the COVID-19 pandemic and the reference years (2017–2019). The darker line illustrates the mean of incidences in the reference years (2017–2019) with confidence intervals

When compared to the reference years, the incidence of all-cause ICU admissions decreased during the lockdown period in 2020 (Fig. 2). The IRR of all-cause ICU admissions was 1.02 (CI: 0.89 to 1.18) before the lockdown in February 2020 when compared to reference years. During the lockdown period in 2020, the IRR of all-cause ICU admissions decreased, falling to 0.78 (CI: 0.67 to 0.90) in March. When the lockdown ended, the incidence rebounded to pre-pandemic levels and remained there until the end of that year. In 2021, the incidence of all-cause ICU admissions remained at the same level when compared to the reference years.

When compared the all-cause ICU admissions in different participating hospitals, the most prominent change in March 2020 was seen in Mikkeli Central Hospital (IRR: 0.69, CI: 0.55–0.86) and in Tampere University Hospital (IRR: 0.78, CI: 0.61-1.00). In Central Finland Central hospital, the incidence of ICU admissions remained higher than in reference years during the year 2021, the IRR being 1.67 (CI: 1.25–2.23) in February 2021 and 1.41 (CI: 1.05–1.89) in September 2021. In Mikkeli Central Hospital, the incidence of ICU admissions was lower than in reference years during 2021. In February 2021, the IRR was 0.84 (CI: 0.68–1.04) and in August 2021, the IRR was 0.86 (CI: 0.70–1.05). In Tampere University Hospital, the incidence remained similar than in reference years during 2021.

The most prominent change occurred in the incidence of diseases of the nervous system (G), the incidence of diseases of the respiratory system (J), and the incidence of neoplasms (C and D) (Table 1). The incidence of the diseases of the nervous system decreased from 30.4 to 100 000 person-years in 2017–2019 to 26.7 per 100 000 person-years (IRR: 0.88 CI: 0.72 to 1.06) in 2020. Thereafter, the incidence increased to 48.1 per 100 000 person-years (IRR: 1.58, CI: 1.34 to 1.87) in 2021 when compared to the reference years. The incidence of diseases of the respiratory system decreased during the study period. The incidence of diseases of the respiratory system was 22.6 per 100 000 person-years during the reference years but decreased to 17.2 per 100 000 person-years (IRR 0.76, CI:0.60 to 0.96) in 2020. In 2021, the incidence was 15.9 per 100 000 person-years (IRR 0.70, CI: 0.55 to 0.89) when compared to the reference years. In the diseases of the blood and neoplasms, the incidence was 84.4 per 100 000 person-years during the reference years and then decreased, being the lowest (73.5) in 2020 (IRR: 0.87, CI: 0.78 to 0.98). The incidence increased slightly in 2021 to 75.8 per 100 000 person-years (IRR: 0.90, CI: 0.80 to 1.01).

**Fig. 3:**
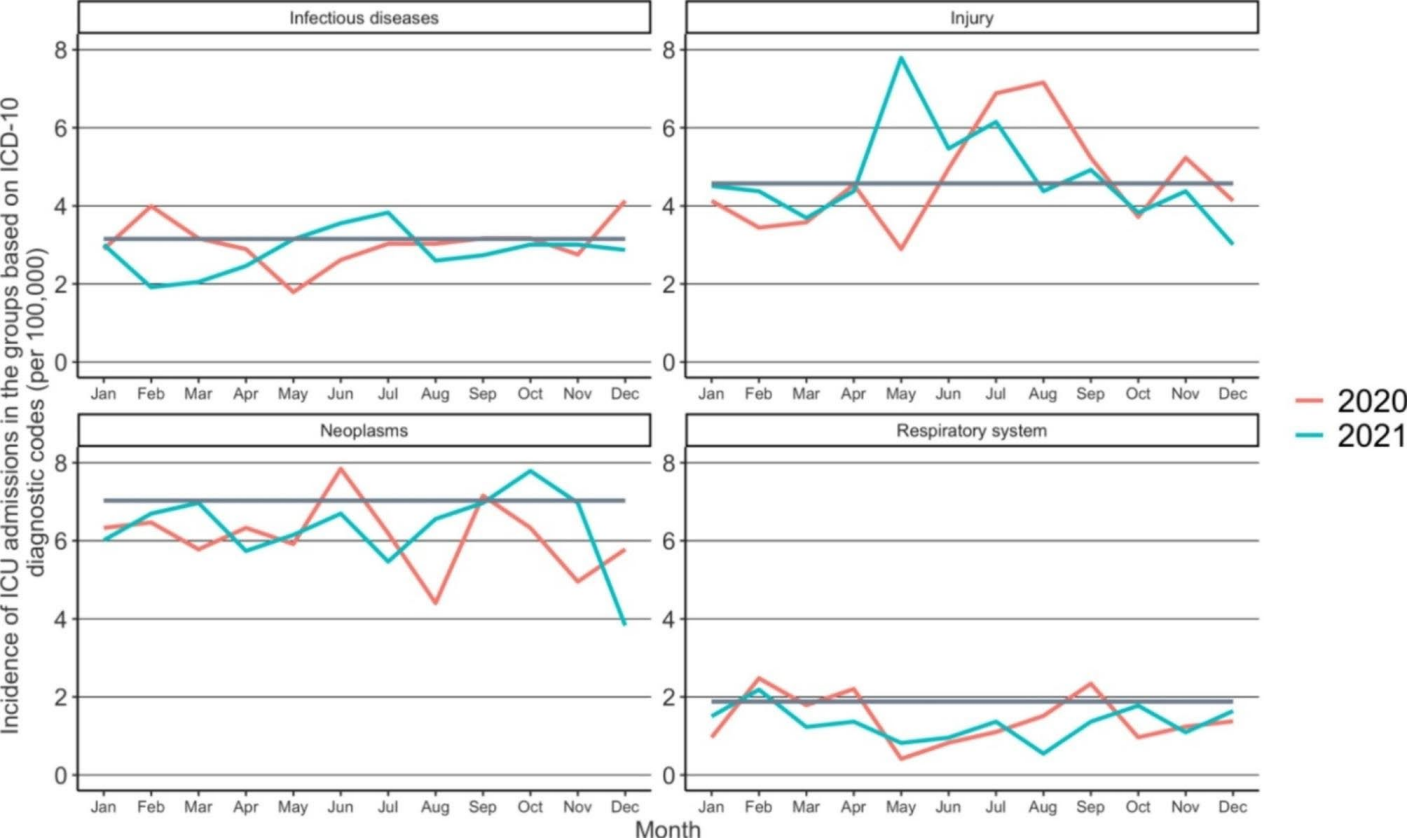
Incidence of ICU admissions in the groups based on ICD-10 diagnostic codes during the COVID-19 pandemic and the reference years (2017–2019). The darker line illustrates the mean of incidences in the reference years (2017–2019)

The incidence of ICU admissions due to trauma (S) remained stable during the years 2020 and 2021 in comparison to the reference years (Fig. 3). During the lockdown in April 2020, the IRR was 1.21 (CI: 0.59 to 2.44). In October, the IRR was 0.81 (CI: 0.41 to 1.61). The incidence of ICU admissions due to trauma in 2021 remained similar to those in the reference years. The IRR was 0.92 (CI: 0.43 to 1.95) in April 2021 and 0.81 (0.41 to 1.60) in October 2021.

There were no changes in the length of ICU stay during 2020 and 2021 when compared to the reference years (Table 2).

**Table 2:** Mean and standard deviation for ICU stay duration in days during the COVID-19 pandemic and pre-pandemic period (2017–2019)

Month	Pre-pandemic 2017–2019		Pandemic 2020–2021	
	Mean	SD	Mean	SD
Jan	1,5	3,3	1,4	2,6
Feb	1,5	3,2	1,5	2,8
Mar	1,6	3,4	1,7	3,7
Apr	1,7	3,3	1,9	4,4
May	1,4	2,8	1,4	2,7
Jun	1,4	2,9	1,4	2,9
Jul	1,5	3,0	1,5	3,1
Aug	1,4	2,8	1,4	2,9
Sep	1,6	3,4	1,4	2,9
Oct	1,5	2,9	1,5	3,0
Nov	1,4	3,0	1,5	3,3
Dec	1,7	4,0	1,7	3,1

## Discussion

The incidence of all-cause ICU admissions decreased after the lockdown was declared in 2020. At the same time, the number of COVID-positive patients in ICU units was highest in April 2020. The proportion of COVID-positive patients admitted to ICU was, however, only 3% in 2020 and 2021. In line with the findings of previous studies, the ongoing social distancing measures and restrictions reduced the spread of other respiratory infections, which are one of the most common reasons for ICU admission [10, 11]. In this study, the most prominent change in all-cause ICU admissions during the lockdown 2020 was seen in Mikkeli Central Hospital, which is a mid-size hospital. The incidence remained lower than in reference years during 2021 in Mikkeli Central Hospital, while an increase was seen in larger Central Finland Central Hospital. Based on the results of the present study, the incidence of severe diseases of the respiratory system in patients requiring ICU treatment decreased during the pandemic, which further supports the findings of previous studies. Moreover, after the announcement of a national lockdown in Finland in 2020, the overall number of emergency department visits and hospital inpatient admissions decreased, which may have also affected the number of ICU admissions [12, 13]. To our knowledge, the effect of the COVID-19 pandemic on the incidence of ICU admissions in Finland has not been previously published.

The number of patients requiring intensive care due to COVID-19 remained low during the pandemic in Finland [2]. This can be explained by the lower incidence of coronavirus in 2020 and 2021 in Finland, compared to many other countries, and the measures implemented, where necessary, during the pandemic.

During the study period, the most remarkable change in ICU admissions occurred in diseases of the nervous system, diseases of the respiratory system, and neoplasms. The incidence of diseases of the nervous system in patients requiring ICU treatment was lowest in 2020 but increased notably in 2021. However, the incidence of neoplasms in patients requiring ICU treatment first decreased in 2020 when compared to the reference years and then increased slightly in 2021. ICU admissions due to neoplasm include patients who have undergone oncological surgery due to malignant tumor. A previous study showed that when the lockdown started in Finland in April, the incidence of oncological surgery decreased slightly but remained at the level of previous years [14]. In addition, caring for COVID-positive patients requires more ICU capacity than patients who do not need isolation. Therefore, the total number of patients treated at the same time in ICUs may have been restricted.

According to previous studies, the number of ED visits due to acute coronary syndrome decreased during the first wave of the COVID-19 pandemic [15–17]. Fear of infection may have caused a decreased willingness to seek treatment and delayed medical intervention may have caused a worsening of the patients’ cardiac disease. This may, therefore, be reflected in an increase in the number of ICU admissions during the second wave of the pandemic.

The incidence of ICU admissions due to trauma remained stable during the pandemic when compared to the reference years. According to a previous Finnish study, the total number of emergency department visits due to injury decreased by 16% during the lockdown period in 2020 [18]. Nevertheless, the incidence of severely injured trauma patients remained unchanged during the first wave of COVID-19 in Finland [19]. The social restrictions and recommendations to stay at home may have reduced the number of minor injuries because of the changes in peoples’ behavior. However, our findings indicate that the rate of severe traumas and patients requiring ICU treatment because of live-threatening injury remained unchanged. According to the previous literature, the number of severe traumas globally decreased or remained stable during the pandemic [20–22].

The strengths of our study include the broad range of data from three large Finnish hospitals. Furthermore, many previous studies have only evaluated the impact of the COVID-19 pandemic in 2020. In this study, we were able to collect follow-up data from all patients during the first two years of the COVID-19 pandemic and to evaluate the impact of the changing restrictions. Our current study also has a limitation that should be addressed. We only analyzed treatment periods according to a patient’s primary diagnosis, and therefore some of the patients may have had more than one diagnosis and reason for ICU stay.

## Conclusions

In conclusion, the incidence of all-cause ICU admissions decreased during the lockdown due to the COVID-19 pandemic. The proportion of patients with COVID-19 requiring intensive care in Finland was only 3% in 2020 and 2021. Restrictions implemented during the pandemic reduced the spread of respiratory infections, which are one of the most common reasons for ICU admission. When the lockdown started in Finland, a slight decrease was seen in the incidence of oncological surgery and the incidence of ICU admissions due to neoplasm decreased. However, the incidence of ICU admissions due to trauma remained stable during the pandemic compared to the reference years.

## Data Availability

Research data are not publicly available due to Finnish research legislation as the Law on the secondary use of routinely collected healthcare data prohibits to share data. Persons interest in gaining access to data must submit official application and study protocol to institutional review boards and after the evaluation of the protocol access to data may be granted, but it must be noted that the current legislation prohibits the delivery of original data out of Finland. The data used in the current study available from the corresponding author on reasonable request.

## Abbreviations

ICU: Intensive Care Unit
CI: Confidence Interval
IRR: Incidence Rate Ratio
IQR: Interquartile Range
